# Development of a deep learning model for intussusception using point-of-care ultrasound

**DOI:** 10.3389/fradi.2026.1812587

**Published:** 2026-07-06

**Authors:** Anand Thyagachandran, Brian Lefchak, Hema A. Murthy, Kelly R. Bergmann, Manu Madhok

**Affiliations:** 1 Department of Computer Science & Engineering, Indian Institute of Technology Madras, Chennai, India; 2 Department of Pediatric Emergency Medicine, Children’s Minnesota, Minneapolis, MN, United States

**Keywords:** deep learning, emergency medicine, intussusception, pediatrics, point-of-care ultrasound

## Abstract

**Objective:**

Intussusception is a pediatric emergency with delays in diagnosis. We sought to develop a novel deep learning model for the detection of target sign on point-of-care ultrasound (POCUS) images.

**Materials and methods:**

POCUS image/video clip media files obtained during emergency department (ED) visits were included for study. Images underwent preprocessing to enhance and resize the region of interest (ROI), ImageNet high dimensional feature extraction and further training using various machine learning models, including classical machine learning, ensemble learning, transfer learning, and fine-tuning. Outputs were analyzed on a patient case, individual image frame and relative threshold bases.

**Results:**

A POCUS image database of originally 785 media files from 49 patients, 8 of whom were positive for intussusception, was converted to 1,582 intussusception and 1,965 normal images for training. The output results show that the fine-tuning models performed better than the classical machine, ensemble and transfer learning models across three different analyses, and that the threshold-based approach to intussusception cases resulted in the greatest predictive performance.

**Discussion:**

Intussusception is a potential candidate for development of deep learning tools due to limited capacity for pediatric focused imaging and accurate diagnosis. Prior studies are limited and have used either large private datasets or formal radiology studies. Modeling involved converting dynamic video files into several static images. Fine-tuning models were best adapted to the screening nature of POCUS images.

**Conclusion:**

Our work demonstrated the feasibility of developing a deep learning model for the detection of intussusception using a smaller dataset of POCUS images.

## 1 Introduction

Intussusception is an emergent medical condition that occurs when a portion of the intestine invaginates into an adjacent section of intestine, leading to blockage and compromised blood flow (1). The condition is most common in infants and young children between 6 and 36 months of age, involving symptoms such as abdominal pain and blood-stained stools (2, 3). Diagnosing intussusception is often difficult because its clinical symptoms overlap with other conditions (4). While non-surgical enema treatment is successful in most cases, delayed treatment can lead to severe and life-threatening complications, such as bowel necrosis or sepsis (1). Thus, prompt identification is crucial as early detection and treatment can significantly improve outcomes and prevent serious complications (2, 3).

**Figure 1:**
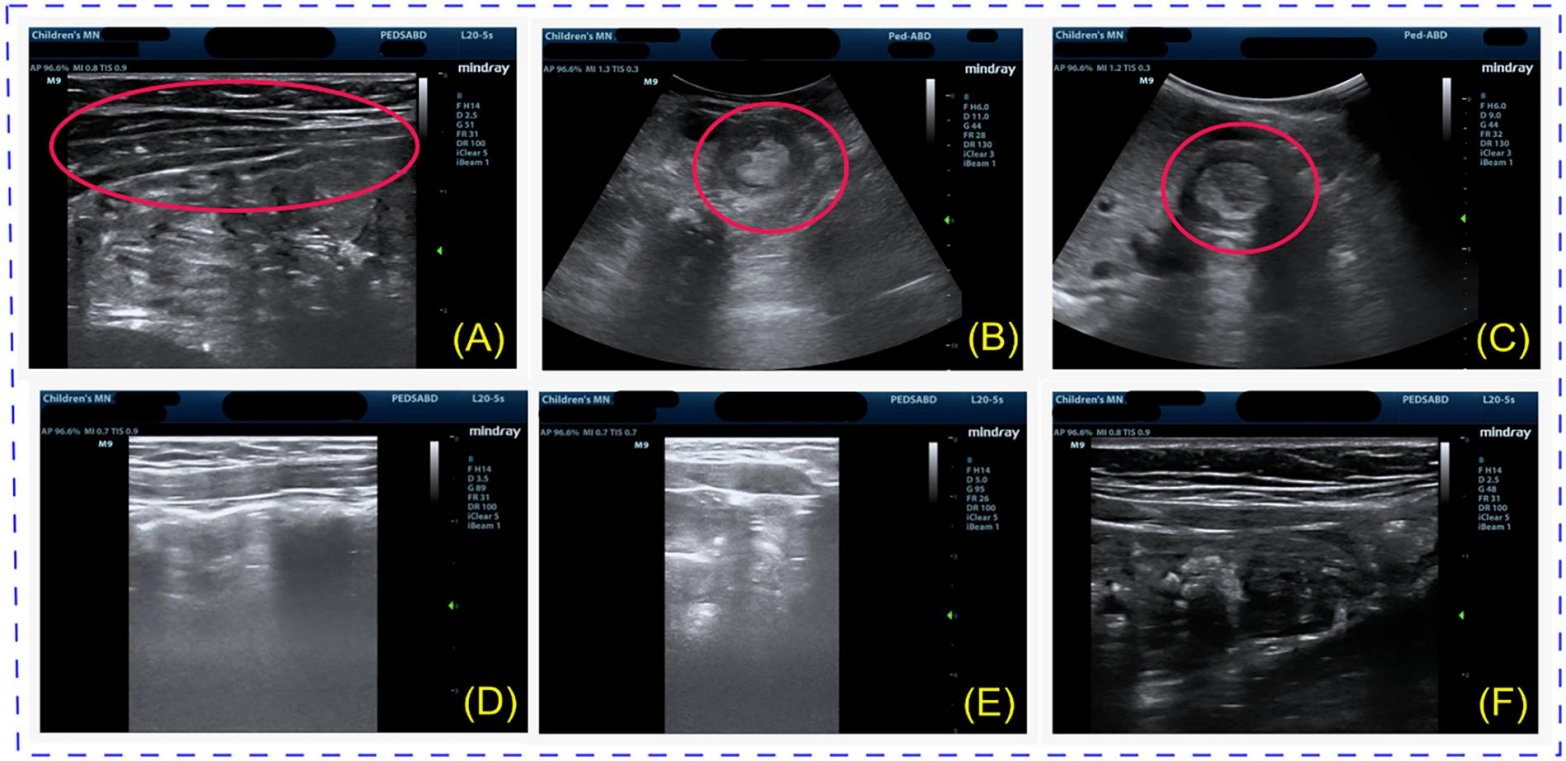
The first row demonstrates point-of-care ultrasound (POCUS) images for intussusception, and the second row demonstrates POCUS images for normal persons. Images A and F demonstrate the longitudinal view, and other images represent the cross-sectional view.

Diagnostic imaging techniques are employed to identify intussusception (1). Ultrasound is considered the first-line diagnostic modality due to its ease of use and high sensitivity and specificity (3, 5). Ultrasound uses non-invasive high-frequency sound waves and spares the use of radiation associated with other imaging modalities (6, 7). Sonographic findings for intussusception include the pathognomonic “doughnut” or “target” sign on cross-sectional view and the “pseudokidney”, “sandwich” or “sleeve” sign on longitudinal view (Figure 1) (2, 8). Despite the beneficial aspects of ultrasound, many facilities still have limited capacity for pediatric imaging or radiologist availability, prolonging the time required for image capture and clinical management, particularly given the majority of pediatric patients are seen outside of children's hospitals in the United States (2, 9–11). Thus, point-of-care ultrasound (POCUS) represents an increasingly utilized technique to expedite ultrasound scans in which the evaluating medical provider performs and interprets the scan entirely at the bedside in real-time (12). POCUS has been shown to be particularly useful within Emergency Department (ED) settings for both screening and diagnostic purposes for many conditions, including intussusception (5, 12, 13). A recent study demonstrated high accuracy and moderate interrater reliability among expert POCUS users for identifying intussusception (sensitivity 94.5% and specificity 94.3%), which is in line with estimates for standard-of-care radiology-performed ultrasound (RADUS); however, sonographer inexperience remains a potential limiting factor to more widespread implementation (14).

Recent advancements in artificial intelligence (AI)-based medical diagnosis using ultrasound and other imaging modalities have garnered significant attention (15–17). These successful attempts have led a few studies to investigate intussusception detection using radiograph or formal ultrasound images with indications of comparative success, such as Li. et al. (sensitivity 92.0% and specificity 94.1%); collectively, however, these works have used large private datasets to train and validate model performance, which may not be readily replicable in other aspects of medical care and may not account for differences related to POCUS images (18–21). AI-assisted technologies developed from official radiology studies may overlook subtle differences related to the distinctive aims of screening and confirmatory modalities. For example, POCUS is often used for screening purposes to determine whether a definitive diagnostic study is subsequently indicated, and thus may entail a different threshold for image capture and interpretation by the end user compared to a more formal study. Furthermore, there may be additional challenges in attempting to utilize dynamic media files, such as video clips which are often obtained with ultrasound, compared to static images (22).

## 2 Objective

Our work therefore sought to demonstrate the feasibility of developing models for the automatic identification of the intussusception target sign using machine learning and deep learning methods based exclusively on POCUS media files obtained as part of routine clinical care and from a smaller dataset compared to previous studies.

## 3 Materials and methods

We have proposed a fully automatic multistage model consisting of a preprocessing stage, feature extraction and classifier model training. The preprocessing stage utilized various image processing algorithms to extract the region of interest (ROI) from the raw image and enhance image quality. ImageNet pre-trained model ResNet50V2 was used to extract high-dimensional feature representation from the preprocessed images (23). Finally, these features were trained further using various standard machine learning models. All study aspects were approved by the respective institutions' Institutional Review Boards (IRBs).

### 3.1 Dataset

The dataset was collected from Children's Minnesota, a large free-standing pediatric health organization in the Midwestern United States with approximately 90,000 ED encounters annually. We selected static (i.e., still frame) and dynamic (i.e., video clip) deidentified abdominal POCUS media files obtained by clinicians with POCUS competency between 2018 and 2020 for the purposes of intussusception detection among children routinely presenting to the ED. As this material was deidentified, additional demographic or clinical features were not included for analysis. These media files were utilized in a separate study on interrater reliability of POCUS and intussusception, and had been originally obtained using a Mindray M9 (Mahwah, New Jersey, United States of America) linear high-frequency transducer via standard scanning technique moving clockwise around the abdomen from an initial right lower quadrant position (14). Patients who had a corresponding positive RADUS scan in addition to POCUS were considered positive for intussusception. Although dynamic ultrasound clips may result in thousands of image frames, only a few frames with the apparent pathological features in view are capable of diagnosing intussusception. The duration of an individual dynamic clip is no more than 6 s with a frame rate of approximately 25 frames per second. Study authors with both clinical pediatric ED and POCUS experience independently labeled individual frames from static and dynamic media files as either intussusception or normal with a third author independently adjudicating any rare discordances. Authors were blinded to the RADUS result at time of labeling. These converted frames were utilized for subsequent models and analysis, an approach used elsewhere in literature (24–26). For the purposes of this feasibility study, we selected the target sign as the defining ultrasound finding for a positive intussusception case. Although there are differences in the sonographic appearance and clinical management of ileocolic and ileoileal intussusception, we did not specify measurement thresholds distinguishing either variant to simplify demonstration of our proof of concept. The training dataset consisted of 1,582 intussusception and 1,965 normal POCUS images. Twenty percent of the training dataset was used to validate the model's performance during training. The robustness of the model was utilized with the test dataset comprising 785 original media files from 49 patients, 8 of whom were positive for intussusception. Frames used for training were not reused for testing and dataset split was performed at the patient level to prevent data leakage.

### 3.2 Preprocessing module

**Figure 2:**
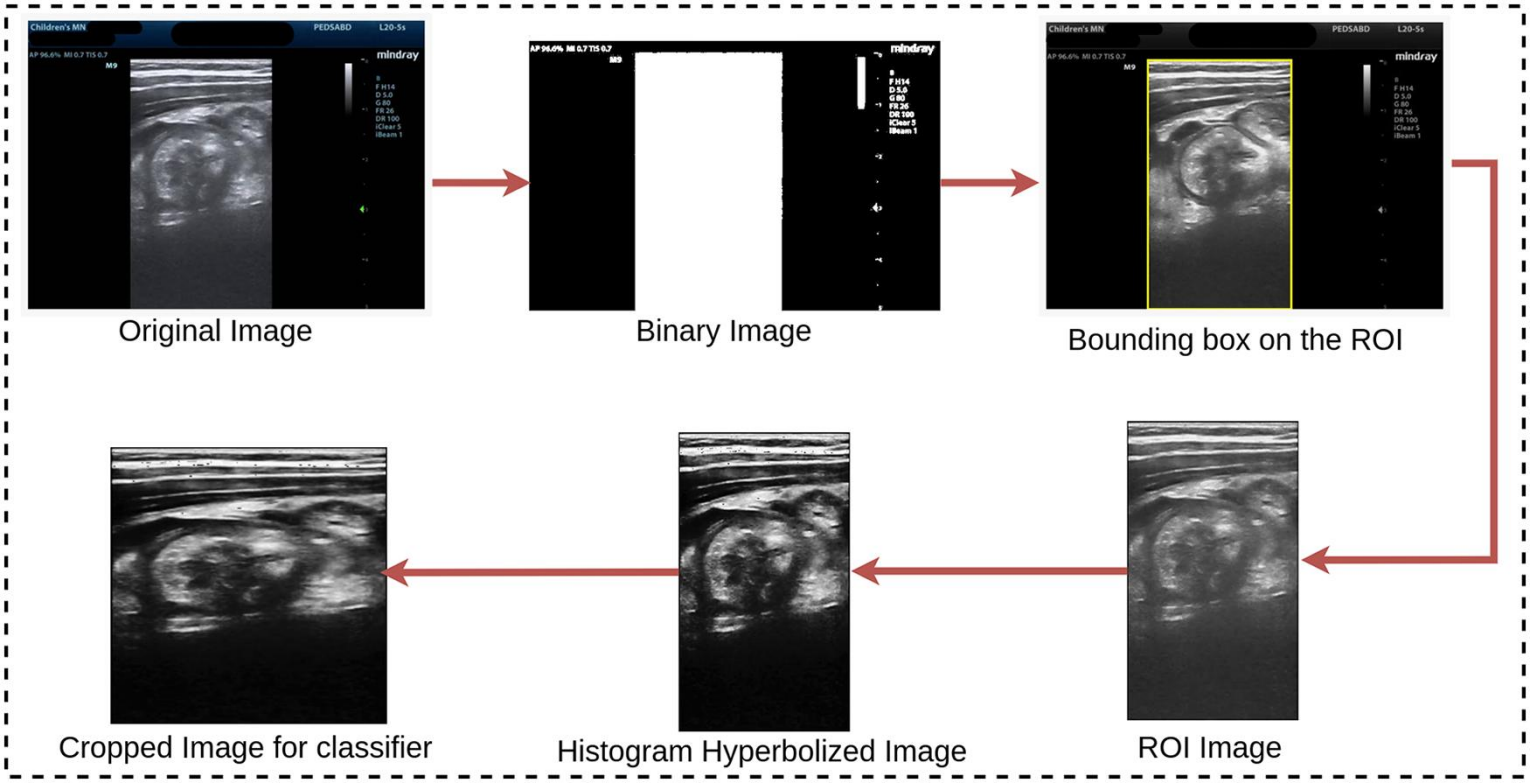
A series of operations are performed in the preprocessing pipeline to extract and enhance the region of interest (ROI).

A series of image processing algorithms were utilized to enhance the ROI in POCUS images and remove unnecessary regions for subsequent analysis (Figure 2). To identify the boundary of the ROI, raw images were binarized. A threshold on the pixel intensity was employed to binarize the input POCUS image. Following binarization, the region props algorithm was applied to identify and cluster connected components. This step provides crucial information about the regions' geometric properties, such as area, centroid and perimeter. Subsequently, a bounding box algorithm was employed to delineate the ROI, allowing for precise localization and isolation of the ROI. Finally, the extracted areas were processed with histogram hyperbolization, an enhancement technique used to optimize contrast and improve the visual quality of the ROI (27). Since the original POCUS images had different dimensions, all images were cropped to (224 × 224) pixels resolution.

### 3.3 Feature extraction module

We used the ImageNet pre-trained model ResNet50V2 to extract high-dimensional feature representation from the preprocessed images (23). This model is highly effective for transfer learning and feature extraction within the field of medical imaging (28–33). Leveraging robust pre-trained weights from ImageNet, this model can be fine-tuned to recognize and classify medical images with high accuracy. The architecture of ResNet50V2 facilitates efficient gradient flow and stable deep network training with its pre-activation residual blocks, which are particularly suitable for the complex and nuanced patterns in medical images (23). When used for transfer learning, the model can quickly adapt to specific medical imaging tasks, such as detecting anomalies in radiographs, classifying tissue types in histopathology images or segmenting organs in magnetic resonance imaging (MRI) scans (23, 34). By extracting high-level features from medical images, ResNet50V2 significantly reduces the need for large annotated datasets, which are often scarce in the medical field. This adaptability and efficiency make ResNet50V2 a powerful tool for enhancing diagnostic accuracy and supporting clinical decision-making processes.

### 3.4 Classification modules and proposed models

**Figure 3:**
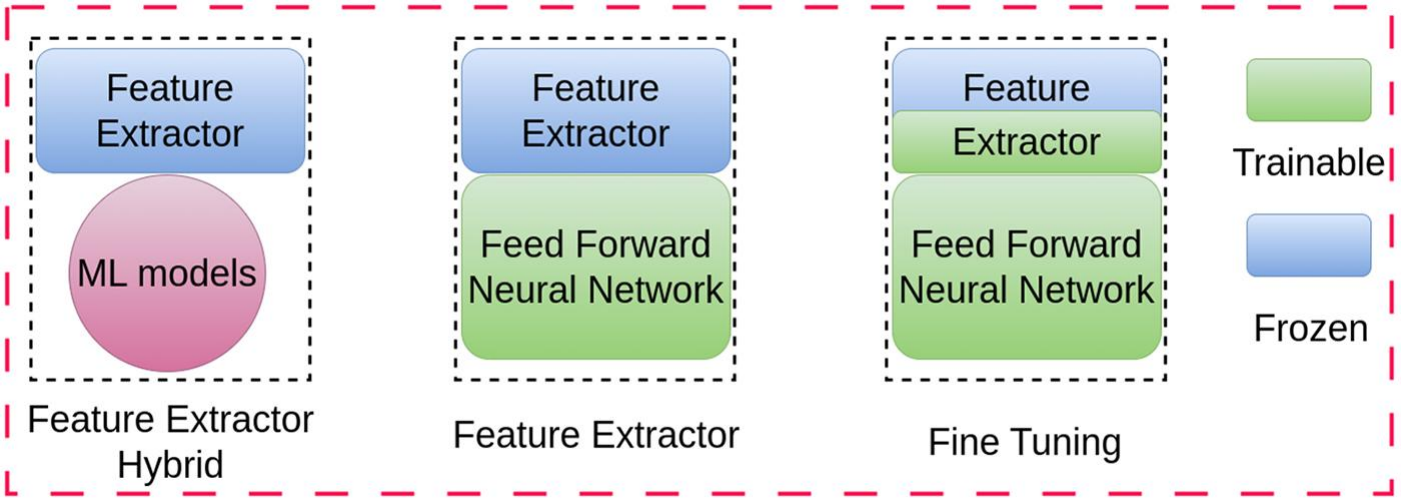
Three different methods to classify the POCUS images.

This work utilized classical machine learning, ensemble learning, transfer learning and fine-tuning models to classify POCUS images into intussusception. Preprocessed images were employed to classify the POCUS images. A simple convolutional neural network (CNN) was considered to be the baseline model. Three techniques were proposed to further classify POCUS images: feature extractor hybrid, transfer learning and fine-tuning (Figure 3). In feature extractor hybrid, the features generated by the ResNet50V2 model are trained with classical machine learning and ensemble learning models. The classical machine learning models, namely Logistic Regression, K-Nearest Neighbour (KNN), Naive Bayes, Support Vector Machine (SVM), and Decision Tree, and ensemble learning models, such as Random Forest, Extreme Boost (XG Boost), Ada Boost, Extremely Randomized Trees Classifier (Extra Tree Classifier), and Gradient Boost are employed in the feature extractor hybrid analysis (35–38). In the transfer learning method, the features generated by the ResNet50V2 model are trained with a shallow neural network. These approaches leverage the rich feature representations learned by ResNet50V2 to enhance the performance of traditional classifiers and neural networks in interpreting ultrasound images.

In the third methodology, the model's adaptability to specific ultrasound imaging tasks is improved by fine-tuning. Specifically, the last few layers of the ResNet50 V2 model are retrained using ultrasound images. This fine-tuning process allows the model to adjust its higher-level feature representations, improving sensitivity to the nuances of ultrasound data and classification accuracy. This approach balances the generalizability provided by the pretrained model with the specificity required for medical image analysis.

### 3.5 Baseline model

**Figure 4:**
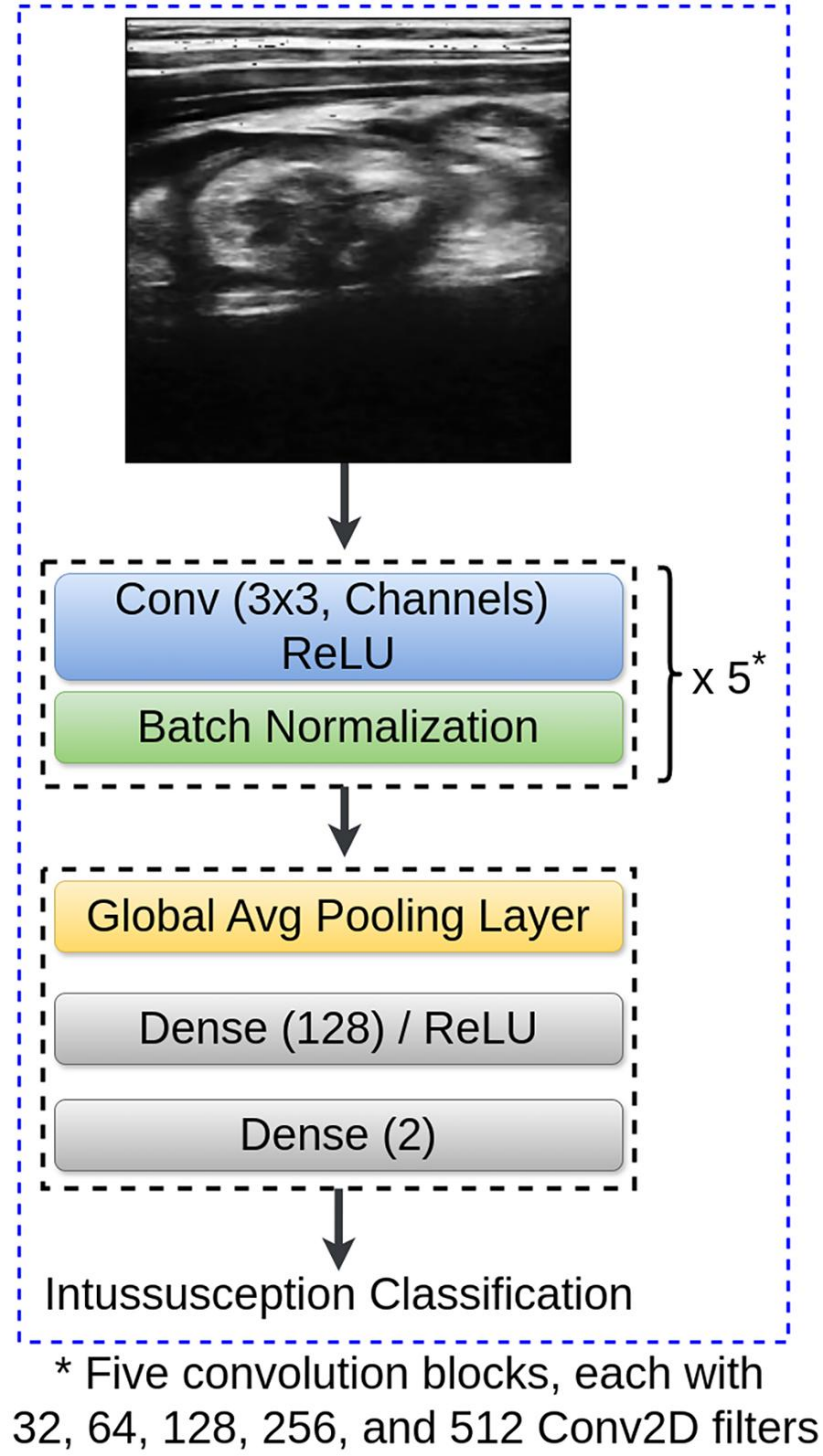
The baseline model, a simple convolutional neural network (CNN) model, consists of five CNN layers followed by batch normalization and finally two linear layers with 128 and 2 neurons, respectively.

A simple CNN model with five layers was considered as the baseline model. Each layer of the baseline model consists of 2D convolution, ReLU activation and batch normalization (Figure 4). Five convolutional layers with 3 × 3 kernel size are employed in each layer. The filters used in each layer are 32, 64, 128, 256, and 512. Batch normalization is employed in each layer to address the issue of internal covariate shift, where the distribution of each layer's input changes during training, slowing down the training process and challenging optimizing the network (39). The final layer feature map is averaged to a vector of dimension 512 and is projected to a dimension of 128 with the feed-forward neural network. Finally, 128-dimensional feature vectors are projected to two-dimensional values, each representing the probability of intussusception.

### 3.6 Experimental settings

Images were reshaped into (224 × 224) pixels before input to the baseline and proposed models. Binary cross entropy was employed as the loss function for the transfer learning and fine-tuning models, which measures the difference between the predicted probability distributions and the actual binary labels (40).

BCE=−(ylog(p)+(1−y)log(1−p))

where:

- y is the true binary label (0 or 1),
- p is the predicted probability of the positive class.

Adam optimizer with a learning rate of 0.0002 was used for model optimization. The model was trained for 15 epochs with a batch size of 60. The parameters of the model were optimized on a validation set. Balanced data from both intussusception and normal classes were used for training. The preprocessing pipeline was written in MATLAB, and the remaining code was written in Python and TensorFlow (41, 42). The machine learning models was implemented using the sklearn package with default hyperparameters (43).

### 3.7 Analysis

Three analytical approaches were performed to facilitate comparing model outputs in the context of real-world POCUS considerations. In the Case Key Analysis, all image frames from patients with positive intussusception were considered positive regardless of visualization of the target sign within the individual frame. This reflects situations in which the POCUS user may not be able to reliably or consistently visualize the target sign in a patient truly with intussusception. In the Image Key Analysis, only the image frames with a visualized target sign were considered positive, while other frames without such visualization were considered normal regardless of the patient's overall intussusception status. Finally, to replicate how some providers may approach interpreting uncertain image findings and given that the total number of POCUS images varies from patient to patient, the Patientwise Analysis utilized a threshold-based analysis of the individual image frames to classify the case as either intussusception or normal. In our study, we empirically chose a permissive threshold value of 30% or greater given the screening nature of many POCUS uses and which we felt best represented real-world practices in our experience. Because the intended use case was screening, the analysis prioritized sensitivity consistent with clinical practice rather than *post hoc* sensitivity analysis optimization. Furthermore, the number of images varied substantially, which limits the interpretability of performance estimates among alternative percentage-based thresholds.

All analyses utilized standard sensitivity, precision, accuracy, and F1 metrics.

## 4 Results

### 4.1 Case key analysis

In this analysis, all image frames from a patient with known intussusception were considered positive, irrespective of the visual findings. Overall sensitivities and precisions for intussusception across the models ranged from 32 to 61 and 36–55, respectively, and model accuracies ranged from 74 to 83 (Table 1). This analysis produced higher sensitivity of baseline model for intussusception than that of other models, but the precision was lowest. Logistic regression and KNN models generally performed better in terms of sensitivity than other classical machine learning, ensemble learning and fine-tuned models.

**Table 1:** Results of the proposed and baseline models based on the case key analysis.

Model	Intussusception		Normal		Accuracy
	Sensitivity	Precision	Sensitivity	Precision	
Baseline	61	36	76	90	74
Logistic Regression	51	41	84	86	78
K-Nearest Neighbour	52	38	81	88	76
Naive Bayes	47	40	84	88	78
Support vector Machine	37	42	88	87	79
Decision Tree	38	42	88	87	79
Random Forest	37	48	91	87	81
Extreme Boost	42	53	92	88	77
Ada Boost	46	39	84	88	77
Extra Tree Classifier	35	49	92	86	83
Transfer Learning	37	48	91	87	81
Fine Tuning Three Layers	37	49	91	87	82
Fine Tuning Five Layers	35	55	94	87	83
Fine Tuning Seven Layers	32	49	93	86	82

### 4.2 Image key analysis

In this analysis, image frames were classified relative to the visual findings specific to each frame, regardless of patient status. Overall sensitivities and precisions for intussusception across the models ranged from 47 to 76 and 20–45, respectively, and model accuracies ranged from 74 to 91 (Table 2). This analysis resulted in generally improved accuracy and sensitivity overall compared to Case Key Analysis. The proposed models, particularly the fine-tuning models, generally performed better than the baseline, classical machine learning and ensemble learning models in terms of precision and accuracy.

**Table 2:** Results of the proposed and baseline models based on the image Key analysis.

Model	Intussusception		Normal		Accuracy
	Sensitivity	Precision	Sensitivity	Precision	
Baseline	74	22	74	97	74
Logistic Regression	76	30	83	97	88
K-Nearest Neighbour	73	26	80	97	79
Naive Bayes	47	20	81	94	78
Support vector Machine	51	28	87	91	84
Decision Tree	53	29	87	91	84
Random Forest	59	38	90	96	88
Extreme Boost	66	35	88	96	88
Ada Boost	66	35	88	98	88
Extra Tree Classifier	66	27	83	96	81
Transfer Learning	64	45	92	96	90
Fine Tuning Three Layers	60	39	91	96	88
Fine Tuning Five Layers	63	41	91	96	89
Fine Tuning Seven Layers	59	44	93	96	91

### 4.3 Patientwise analysis

In this analysis, a threshold of 30% or more image frames with visual findings consistent with intussusception was required for the respective case to be considered positive, taking into consideration real-world practices and the varying number of POCUS scans among cases. Overall sensitivities and precisions for intussusception across the models ranged from 38 to 88 and 38–71, respectively, and model accuracies ranged from 76 to 90 (Table 3). The fine-tuning models generally performed better than baseline, classical machine learning and ensemble learning models in terms of sensitivity, precision and accuracy.

**Table 3:** Results of the proposed and baseline models based on the patientwise analysis.

Model	Intussusception		Normal		Accuracy
	Sensitivity	Precision	Sensitivity	Precision	
Baseline	75	38	76	94	76
Logistic Regression	75	43	80	94	88
K-Nearest Neighbour	88	47	80	97	82
Naive Bayes	62	38	80	92	78
Support vector Machine	38	50	93	88	84
Decision Tree	50	50	90	90	84
Random Forest	62	45	85	92	82
Extreme Boost	88	54	85	92	82
Ada Boost	75	43	80	94	80
Extra Tree Classifier	50	50	90	90	84
Transfer Learning	62	62	93	93	88
Fine Tuning Three Layers	62	62	93	93	88
Fine Tuning Five Layers	62	71	95	93	90
Fine Tuning Seven Layers	75	55	88	95	86

### 4.4 Comparative analysis

**Figure 5:**
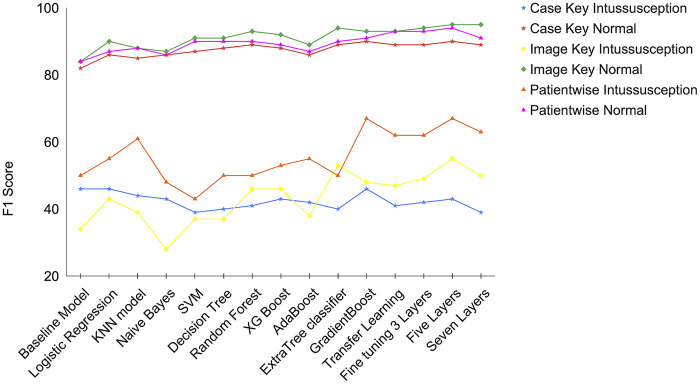
F1 score results of the baseline and proposed models with the test dataset.

F1 score trends of the baseline and proposed models demonstrated improved performance of the fine-tuning models compared to the other baseline, classical machine learning and ensemble learning models for both Image Key and Patientwise Analyses (Figure 5).

## 5 Discussion

Our study demonstrated the feasibility of developing deep learning models for the identification of intussusception using non-RADUS POCUS images from a small medical dataset. Fine-tuning models performed better than classical machine, ensemble and transfer learning models and a threshold-based approach to intussusception cases resulted in the greatest predictive performance. The results of our study have implications for those interested in the integration of AI-assisted technology, particularly POCUS for clinical purposes and future real-time diagnosis.

Recent technological advancements in AI-based medical diagnosis within imaging modalities have garnered significant attention (15–17). The inherent capability of deep learning methods to quantify patterns from complex data holds promise for numerous future applications within many subfields of radiology, including detection assistance and efficiency improvement (44, 45). While challenges exist in leveraging such technologies toward practical implementations and point-of-care uses, interest in AI-assisted POCUS represents a noteworthy example of potential cutting edge applications assisting practitioners at the bedside (46).

Previous studies have used radiographs and ultrasound images for intussusception detection, typically by demarcating the ROI within bounding boxes (18–20). To our knowledge, Li et al. first proposed an enhanced Faster R-CNN deep learning framework consisting of three stages: a backbone for feature extraction network, region proposal network (RPN) and ROI pooling (18, 47). VGG16 extracts feature maps from an input image, the RPN generates the ROIs with the help of Nonmaximum Suppression (NMS) and finally projects the ROIs to a feature map (48). Pei et al. and Kim et al. used the YOLOV5 model from ultrasound images (19, 20). YOLO integrates region proposal, feature extraction, classification, and bounding box regression into a single stage (49). Chen et al. proposed an end-to-end model, the Children Intussusception Diagnosis Network (CIDNet) system, a transformer-based model which utilizes the embeddings generated by the EfficientNetB7 model and classified ultrasound images into target sign, “pseudokidney” sign or normal from ultrasound images (21, 50).

While collectively these works have used large annotated datasets to train and validate model performance, such datasets may not be readily replicable in other aspects of medical care (18–21). Additionally, we were interested in exploring model development from specifically POCUS images, given its increasing utilization and unique point-of-care nature. We therefore utilized a much smaller dataset of exclusively POCUS images originally obtained and interpreted at the bedside, and successfully employed image processing algorithms and fine-tuning of deep CNN models trained with the ImageNet database to extract high-dimensional features followed by machine learning models and feed-forward neural networks (34). Using various models, we analyzed and reported on three general approaches to classifying intussusception cases. We believe these different approaches reflect real-world differences providers may employ when interpreting POCUS scans. For example, some providers might deem a case positive based on a single high-quality image, whereas others may prefer having multiple image frames before reaching a decision, particularly if the images are of uncertain or lower quality. Collectively, our output results show that the fine-tuning models perform better than other models, and that the threshold-based approach resulted in the greatest predictive performance. Threshold aggregation may reduce the impact of image-level label noise, which seems likely often given the presence of many non-diagnostic frames. While not always true for other applications, the fine-tuning models likely performed better in our study because the pretrained ResNet50V2 model adapts specifically to the ultrasound dataset. By retraining the last few layers, the model can adjust to the unique patterns and nuances in POCUS images, leading to improved feature representation and, consequently, higher classification accuracy. This approach enhances the model's sensitivity to the subtle differences between intussusception and normal cases, which appears further augmented by the parameters of a threshold-based approach to case classification. Given the limited existing literature on this topic at present, external performance comparisons with other ResNet50V2 models are restrained, limiting comparative benchmarking.

Our study results provide relevant insights during a time of growing interest in deep learning applications within medical imaging. Our output results favoring fine-tuning models of a threshold-based approach may offer further considerations for other efforts utilizing screening-based image modalities. We consider the use of POCUS images to be a unique asset of our study as they were obtained at the bedside and provide a potential early diagnosis and differentiation of ileocolic from ileoileal intussusception. Our experience also offers insights for similar endeavors utilizing smaller datasets. Furthermore, the feasibility of AI-assisted POCUS techniques such as the one evaluated in our study highlights the potential role of such technologies within pediatrics and the unique considerations in optimizing safe and effective imaging utilization for this particular population (51). We envision a future in which embedded AI-assisted image recognition tools augment bedside decision-making, enabling earlier referral for definitive imaging and supporting inherently challenging transfer decisions for the many children that initially present to institutions without pediatric ultrasound or intussusception treatment capabilities. Additionally, such technologies could be leveraged to enhance radiology workflows by assisting in the flagging of abnormal images.

Our study has several limitations, most notably the small sample size and single institution setting. Although the dataset was expanded by extracting individual frames from dynamic clips, this approach raises the possibility of overfitting or limited generalizability due to correlation among frames. However, POCUS is inherently dynamic, and sequential frames frequently represent distinct imaging planes with varying anatomical features entering and exiting the field of view—indeed, an aspect of POCUS that itself contributes to the challenge of image interpretation for clinicians. Moreover, intussusception is characterized by a relatively constrained and well-described sonographic appearance, which limits pathological heterogeneity. Our study also did not stratify cases by the two main intussusception subtypes and their associated quantitative measures, thereby restricting the model's ability to rely on patient- or episode-specific features. Taken together, while these factors remain important considerations for future work, they collectively reduce the likelihood that models simply memorized patient-specific or institution-specific characteristics rather than learning generalizable visual patterns. While a more granular qualitative failure analysis may still be highly valuable for understanding model limitations, this was not built into our initial proof-of-concept protocol and several standardizing aspects were taken into consideration to limit data heterogeneity.

As noted above, partly due to our focus on demonstrating feasibility of concept, our investigation did not encompass longitudinal sign or measurement differences between ileocolic and ileoileal intussusception. Future work could expand analysis to include these aspects as well as expanding sample size and drawing comparisons to human performers to further assess the incremental value of such AI tools. In particular, a comparative assessment across POCUS users with different experience levels would be especially informative. Additionally, future work could investigate the performance of AI-assisted technology during real-time use within POCUS machines.

## 6 Conclusion

Our study demonstrated the feasibility of developing machine learning and deep learning models for the identification of intussusception using POCUS images from a small medical dataset. Fine-tuning models performed better than classical machine, ensemble and transfer learning models. Our study results have implications for those interested in the integration of AI-assisted technologies within POCUS imaging for aiding real-time diagnosis.

## Data Availability

The datasets presented in this article are not readily available because dataset images are not permitted for further disbursement beyond the confines of those included for the study conduction. Requests to access the datasets should be directed to Manu Madhok, manu.madhok@childrensmn.org.
